# Volatile Compounds, Sensory Profile and Phenolic Compounds in Fermented Rice Bran

**DOI:** 10.3390/plants10061073

**Published:** 2021-05-27

**Authors:** Ardiansyah, Annisa Nada, Nuraini Tiara Indah Rahmawati, Annisa Oktriani, Wahyudi David, Rizki Maryam Astuti, Dody Dwi Handoko, Bram Kusbiantoro, Slamet Budijanto, Hitoshi Shirakawa

**Affiliations:** 1 Department of Food Technology, Universitas Bakrie, Jakarta 12920, Indonesia; nadannisa@gmail.com (A.N.); nuraini.tiara@gmail.com (N.T.I.R.); annisa.oktriani@gmail.com (A.O.); wahyudi.david@bakrie.ac.id (W.D.); rizki.astuti@bakrie.ac.id (R.M.A.); 2 Laboratory of Flavor Analysis, Indonesian Center for Rice Research, Indonesian Agency for Agricultural Research and Development, Ministry of Agriculture, Subang, Jawa Barat 41256, Indonesia; dodyhandoko@gmail.com (D.D.H.); hbram_kusbiantoro@yahoo.com (B.K.); 3 Department of Food Science and Technology, Faculty of Agricultural Engineering and Technology, IPB University, Bogor 16680, Indonesia; slametbu@apps.ipb.ac.id; 4 Laboratory of Nutrition, Graduate School of Agricultural Science, Tohoku University, 468-1 Aramaki Aza Aoba, Aoba-ku, Sendai 980-8572, Japan; shirakah@tohoku.ac.jp; 5 International Education and Research Center for Food Agricultural Immunology, Graduate School of Agricultural Science, Tohoku University, Sendai 980-8572, Japan

**Keywords:** antioxidant activity, fermented rice bran, rice bran, sensory profile, volatile compounds

## Abstract

Rice bran (RB), a by-product of the rice milling process, is a rich source of bioactive compounds. Current studies have suggested that fermentation can enhance the bioactivities of RB. This study is aimed to analyse the volatile compounds and sensory profile of fermented RB from two cultivars (Inpari 30 and Cempo Ireng) that are well-known in Indonesia, as well as to measure total phenolic content (TPC) and antioxidant activity. Volatile compounds of fermented RB were analyzed using gas chromatography-mass spectrometry combined with headspace-solid phase microextraction. The optimum TPC and antioxidant activity were observed after 72 h fermentation of RB. The 55 volatile compounds were identified in fermented and non-fermented RB. They were classified into alcohols, aldehydes, acids, ketones, phenols, esters, benzene, terpenes, furans, lactone, pyridines, pyrazines, and thiazoles. Volatile compounds were significantly different among the varieties. The sensory analysis showed that the panelists could differentiate sensory profiles (color, taste, flavor, and texture) between the samples. Fermentation can enhance the acceptance of RB. These studies may provide opportunities to promote the production of fermented RB as a functional ingredient with enhanced bioactivity for health promotion.

## 1. Introduction

Rice has been cultivated in the South East Asia region, including Indonesia, since 2500 B.C. [1]. There are several varieties of rice in Indonesia such as aromatic, non-aromatic, and pigmented. Indonesia produced around 54.60 million metric tons of rice in 2019 [2]. Polished rice is the major product of the rice milling process, with 8–12% being the by-product rice bran (RB); around 5.5 million metric tons of RB were produced in the same year.

RB is a by-product of rice milling, sitting between the between endosperm and the outer layer of rice. RB has received much attention because it contains diverse active compounds that have a broad spectrum of health benefits [3,4,5,6]. These properties are ascribed to the high amounts of total flavonoid, tocopherols, tocotrienols, γ-oryzanol, and total phenolic content (TPC) [7,8]. Phenolic compounds are key compounds in antioxidant activities due to their capacity to scavenge free radicals, disrupt radical chain reactions, and chelate metal ions [9]. In some pigmented rice cultivars, the pigment is concentrated in the outer layer of the rice grain. Pigments in the outer layers are responsible for the color of some rice cultivars which are referred to as pigmented cultivars. In Indonesia, pigmented rice is classified into black and red rice.

Solid-state fermentation (SSF) using microorganisms and their enzymes is one of the beneficial strategies to increase bioactive compounds in plant foods [10]. SSF is effective at increasing TPC, antioxidant activity, and organic acid [11,12,13]. The activity of the enzyme β-glucosidase in fungi increases hydroxyl compounds that increase free phenolic compounds in RB [14]. Recently, our groups’ studies have shown that SSF with *Rhizopus oligosporus* can induce enzyme activity that could also lead to the release of phenolic compounds, higher antioxidant activity, blood pressure lowering activity in stroke-prone spontaneously hypertensive rats, anti-α-amylase activity, and antiproliferative properties toward colon cancer in WiDr cell lines [15,16,17].

Volatile compounds are chemicals that play a key role in the formation of aromas in food products. The volatile compounds of RB have been reported by several investigators. They have used gas chromatography-mass spectrometry (GC-MS) to identify the compounds. The identified compounds of RB consist of alcohol, alkanes, ketones, and aldehydes [18,19,20]. Hexanal is a common compound in RB that may cause off-odor in RB because of enzyme activity during processing of RB [21,22]. Another report has shown that volatile compounds of black RB consist of aldehyde, alcohol, alkenes, and ketone [21]. Current data shows that volatile compounds of fermented RB by different lactic acid bacteria were acids, aldehydes, esters, furan derivatives, ketones, alcohols, benzene and benzene derivatives, hydrocarbons, and terpenes [23]. Furthermore, when RB was fermented by *Lactobacillus paracasei*, it produced some volatile compounds, especially lactones, 2,3-butanedione, and 3-hydroxy-2-butanone, which are similar to a compound in dairy products (i.e., cheese, fermented milk, and butter). These can improve the sensory profile of rice-based probiotic functional foods [24,25].

This is the first study to investigate the formation of volatile compounds in Indonesian fermented RB. The objective of this study was to analyze the profiles of volatile compounds of two cultivars of RB in Indonesia as well as TPC and antioxidant activity. Inpari 30 (IPR30) cultivar is one of the Ciherang cultivars (white rice) that is most widely consumed in Indonesia. Cempo Ireng (CI) black rice is one of the local pigmented rice varieties in Indonesia and has a high content of phenolics, total flavonoids, and total anthocyanins [16]. In this study, new methods have been also developed to identify the sensory profile of fermented RB using projective mapping (napping) methods. Napping is a fast sensory-analysis method based on the spontaneous placement of products by panelists conducted in two dimensions by grouping the same attributes [26].

## 2. Results

### 2.1. Total Phenolic Content and Antioxidant Activity

The TPC was significantly higher (*p* < 0.05) in both fermented RB after 72 and 96 h of fermentation than in non-fermented RB (0 h) (IPR30; 1.57 ± 0.19 and CI; 6.12 ± 0.70 mg GAE/g dry basis (DB), respectively. However, fermentation for 24 h is not sufficient to increase the TPC of fermented RB (Table 1). The highest TPC was obtained at 72 h fermentation of IPR30 and CI fermented RB (2.24 ± 0.21 and 7.85 ± 0.62 mg GAE/g DB, respectively (Table 1). 

The fermentation process significantly increased in (*p* < 0.05) the DPPH RSA of both cultivars, with the highest RSA observed after 96 h of fermentation at the level 69.50 ± 1.53% and 48.12 ± 2.84%, respectively (Table 1). However, prolonging of the incubation time until 96 h was not significantly different when compared with 72 h of fermentation of RSA and TPC, respectively; then, we decided to use both varieties with 72 h fermentation for further analyses. 

### 2.2. Volatile Compounds of Rice Bran

**Figure 1:**
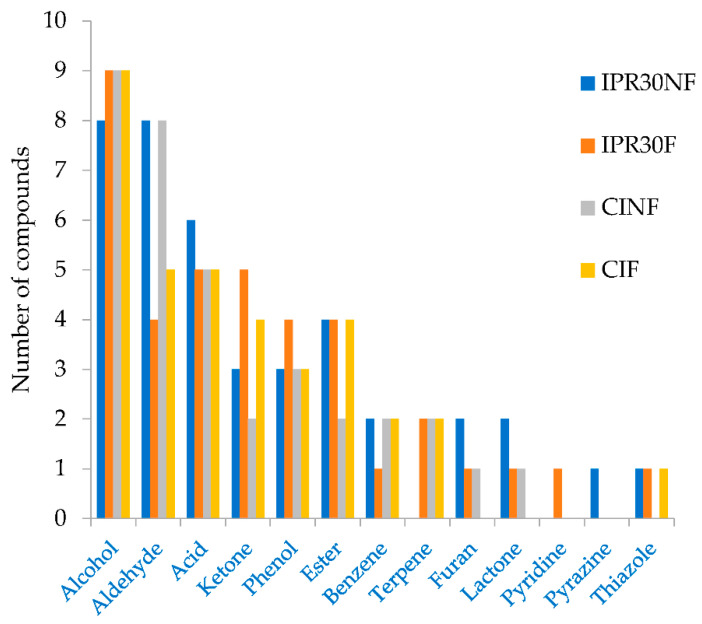
Groups of volatile compounds in fermented or non-fermented rice brans. Inpari 30 non-fermented (IPR30NF); Inpari 30 fermented (IPR30F); Cempo Ireng non fermented (CINF); and Cempo Ireng fermented (CIF).

The volatile compounds of fermented and non-fermented RB of IPR30 and CI were shown in Table 2. The 55 identified volatile compounds consisted of 13 alcohols, 11 aldehydes, 6 acids, 5 ketones, 4 phenols, 4 esters, 2 benzene, 3 terpenes, 2 furans, 2 lactones, 1 pyridine, 1 pyrazine, and 1 thiazole. The alcohol compounds were the most volatile compounds that were detected in both RB varieties (fermented and non-fermented), followed by aldehydes, acids, ketones, phenols, esters, benzenes, terpenes, furans, pyridines, and thiazole (Figure 1).

**Figure 2:**
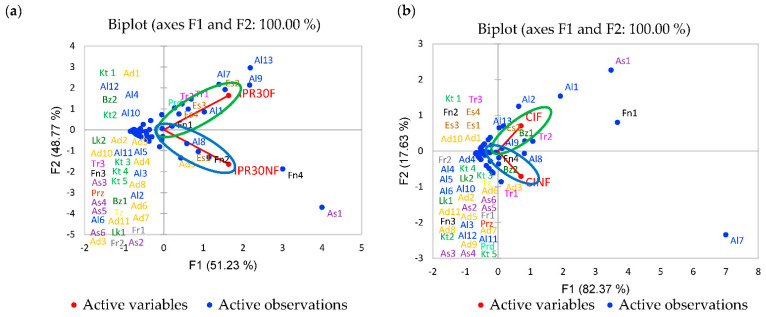
PCA plot of volatile compounds of (**a**) Inpari 30 (IPR30) and (**b**) Cempo Ireng (CI) (F, fermented; NF, non-fermented).

The present study applied principal component analysis (PCA) to compare the differences and identify dominant volatile compounds among the fermented and non-fermented RB. The PCA plot of volatile compounds in Figure 2 shows RB with fermentation and without fermentation located in different dimensions. It shows that the fermentation process can affect the volatile compounds of RB.

Volatile compounds of IPR30 fermented RB were dominated by 3-methylbut-3-en-1-ol; 2,3-butandiol; benzylalcohol; glycerin; methyl hexadecanoate; (E)-9-methyl octadecanoate; (Z, Z) -9,12-methyl octadecadienoate; 1R-alpha-pinene; caryophyllene; 2-methoxyphenol; and 3-methyl pyridine. Most of these compounds were formed from lipid oxidation through enzymes activity in the mold that was used as the starter of fermentation. During the sterilization process in preparation of RB before fermentation, 2-Methoxyphenol and 3-methylpyridine were formed as a product of the Maillard reaction. These compounds contributed to sweaty, creamy, fatty, pungent, and smoky aromas. Conversely, IPR30 non-fermented RB was dominated by 2-furanmetanol; nonanal; methyl tetradecanoate; phenol; and 4-ethenyl-2-methoxyphenol (Figure 2a). These compounds contributed to burnt, nutty, and fatty aromas.

Meanwhile, the volatile compounds of CI fermented RB were dominated by 4-methyl-3-penten-1-ol; benzyl alcohol; glycerin; methyl hexadecanoate; ethylbenzene; and caryophyllene. Most of these compounds were formed by hydrolysis of fatty acids in RB through enzyme activity in the mold that was used as the starter for fermentation. Furthermore, CI non-fermented RB was dominated by 2-furanmetanol; hexanal; naphthalene; 1R-alpha-pinene; and 4-ethenyl-2-methoxyphenol (Figure 2b) that contributed to burnt, nutty, fatty, and pungent aromas.

### 2.3. Sensory Profile of Rice Bran

**Figure 3:**
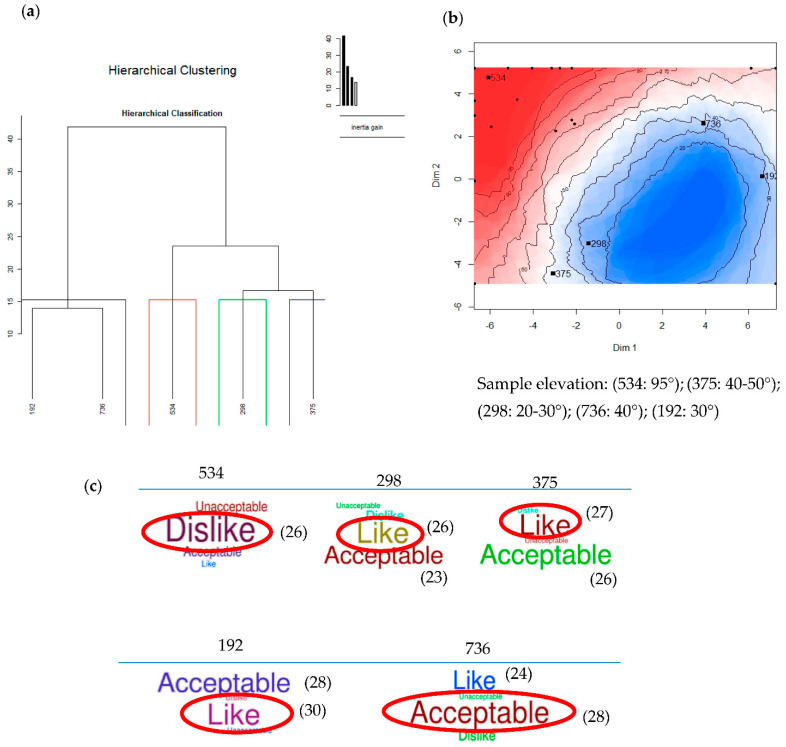
(**a**) Hierarchical Clustering Map; (**b**) Preference Mapping; and (**c**) Overall Acceptance (frequency panelist) of fermented and non-fermented rice bran. 534: Benchmark (Control), 192: CIF, 736: CINF, 298: IPR30F, and 375: IPR30NF.

A hierarchical clustering map was calculated based on the distance between the samples. The samples with a close distance were grouped as the same cluster. Figure 3a showed that there were two clusters. The first cluster consisted of samples 192 and 736; both samples were derived from CI fermented and non-fermented RB, respectively. The second cluster consisted of samples 298, 375, and 534. These samples, clustered into a single group, were derived from Inpari 30 fermented RB, Inpari 30 non-fermented RB, and benchmark, respectively.

Enhancing knowledge of consumer preference, we continued to analyze preference mapping of RB as shown in Figure 3b. The overall liking of the samples and sensory experiences has an important factor that contributes to consumers’ buying expectations and decisions to buy products in the market. The data of overall acceptance (n = 75) of fermented and non-fermented RB are shown in Figure 3c.

## 3. Discussion

Phenolic compounds are widely found in plant products and they have antioxidant properties [10]. Our results showed that an increase in RSA was related to an increase in TPC, suggesting that TPC is responsible for antioxidant activities. *Rhizopus oligosporus* have been reported to be able to produce enzymes such as β-glucosidase, amylase, cellulase, chitinase, inulinase, phytase, xylanase, tanase, esterase, invertase or lipase that can enhance bran cell wall degradation, thus producing more free phenolic compounds. In addition, several studies have reported that phenolic acids, flavonoids, anthocyanins and others also contribute to the antioxidant activity of RB [27,28,29]. Another study showed that during fermentation, there was an increase in the content of chlorogenic acid, p-hydroxybenzoic acid, gallic acid, and ferulic acid vanillin in RB [10].

In this study, ethanol was significantly higher in most of the fermented RB. Ethanol is mainly formed from the fermentation process, and is derived from pyruvate through acetyl-CoA, whereas it is derived from the pentose phosphate pathway of glucose [23]. 3-methyl-3-buten-1-ol and 2-ethyl-1-hexanol contribute to sweaty, fruity, citrus, and oily aromas, respectively. Linalool was only found in CI non-fermented RB and contributed to citrus, greeny, and waxy aromas. Benzyl alcohol are formed via reduction of benzoic acid during fermentation through the glycolysis pathway [30] was higher in fermented RB. Phenylethyl alcohol are formed via hydrolysis of phenylethyl acetal and phenylethyl ester contributed to aroma floral and slightly rose [31,32]. Eugenol was found in all samples of fermented and non-fermented RB. Eugenol contributed to a sweet, clove-like, green aroma [33]. 2,3-butandiol, 3-methyl-3-butenol, benzyl alcohol and 2-furanmetanol are the main alcohol compounds and were formed in both IPR30 and CI fermented RB, including in CI non-fermented RB.

Acids are formed through the pentose phosphate pathway and the TCA cycle [34]. Furthermore, the acid group in this study was probably derived from aldehyde oxidation and lipid hydrolysis [35]. The present study showed that acetic acid produced a sour aroma in RB (Table 2). Hexanoic and octanoic acids contributed to sweaty and cheesy aromas (Table 2), while nonanoic acid contributed to greeny and fatty aromas (Table 2) [22,36]. The relative area of acetic acid is seen at higher levels in IPR30 non-fermented and CI fermented RB (Table 2). Acetic acid is also known to originate from the oxidation of acetaldehyde [34].

Ketones were detected in fermented RB, such as butenone, 4-hydroxybutan-2-one, 1-(1H-pyrrol-2yl) ethanone, 2-pyrrolidinone, and 6,10,14-trimethyl-2-pentadecanone. Ketones can give a pleasant aroma [37]. They can produce caramel, nutty, walnut, bready, oily, and herby aromas (Table 2), and are formed from fatty acids by enzymatic oxidative decarboxylation [21,22].

Esters, which have a fatty, waxy, and oily aroma (Table 2), are formed by esterification between alcohols and acids in fermentation [22]. The current study determined four esters, methyl tetradecanoate, hexadecanoic acid methyl ester, methyl (E)-octadec-9-enoate, and methyl (9Z,12Z)-octadeca-9,12-dienoate, in the fermented RB samples (Table 2), although they were present at low concentrations. The compounds of methyl (E)-octadec-9-enoate, and methyl (9Z,12Z)-octadeca-9,12-dienoate were not detected in CI non-fermented RB.

An interesting finding of the present study was that 2-pentylfuran, which has a greeny, beany, and buttery aroma (Table 2), has been reported as one of the odor-active compounds in various rice cultivars [22]. This compound was only detected in non-fermented IPR30. 2-methoxyphenol was significantly higher in CI RB (fermented and non-fermented) than IPR30 (Table 2), which contributes to the aroma in black rice [20].

We found that both samples 192 and 736 (Cempo Ireng fermented and Cempo Ireng non-fermented RB) were located in one cluster and have similar characteristics in taste attributes (savory, sweet, and salty), color attributes (black), and aroma attributes (fresh and milk) (Figure 3a); however, even though they have similar characteristic, 192 and 736 have a different elevation value of preference (Figure 3b). The second cluster consists of samples 298, 375, and 534 (Inpari 30 fermented, non-fermented, and benchmark, respectively) which were clustered into single group because they have similar characteristics in taste attributes (sweet, bitter, and savory), color attributes (yellow), and aroma attributes (fresh, milk, and rice).

Sample 534 has the highest elevation value (95°) because the benchmark (control) was derived from white rice RB, followed by sample 375 (IPR30NF) with an elevation value of 40–50° and sample 298 (IPR30F) with an elevation of 20–30°. As they were located in the same dimension, sample 375 has similar sensory characteristics to sample 298 as both samples were IPR30 RB. Furthermore, samples 192 (CIF) and 736 (CINF) were both located in the same dimension with an elevation value of 30° and 40°, respectively. Both samples have similar characteristics as they are similar varieties of CI RB.

Panelists could not accept or dislike (n = 26) sample 534 (benchmark) because of its taste (bland, pungent, rancid, and bitter) and aroma (bland, pungent, bitter rancid, and sour). Conversely, panelists liked and accepted samples 298 (IPR30F) (n = 26 and 23); 375 (IPR30NF) (n = 27 and 26); 192 (CIF) (n = 28 and 30); and 634 (CINF) (n = 24 and 28) as these samples had the dominant and accepted taste (sweet and savory) and aroma attributes (milk, sticky rice, and fresh). These results were consistent with data in preference mapping (Figure 3b). Positions of samples 192 (CIF), 736 (CINF), 298 (IPR30F) and 375 (IPR30NF) were at different poles and colors (blue and degraded blue) when compared to sample 534 (benchmark or control) at the red pole. Samples at the blue pole had been liked, accepted, and preferred by the panelists, while the samples at the red pole were not liked or preferred.

In this study, SSF is shown to increase the bioactivity of rice bran, similar to results shown with our previous study (15–17). Other researchers have also shown the same phenomena (10–13). SSF is one strategy to improve the sensory profile of rice bran, although future studies are needed to expand the study with a greater number of cultivars of rice. Fermentation can increase the functional properties of RB, in order to use RB for functional ingredients, and the creation of novel, functional food for prolonging healthy life.

## 4. Materials and Methods

### 4.1. Chemicals and Reagents

Methanol, Folin Ciocalteu’s phenol reagent, gallic acid, 2,2-diphenyl-1-picrylhydrazyl (DPPH), and 2,4,6-trimethylpyridine were purchased from Sigma-Aldrich Co. (Saint Louis, MO, USA). *R. oligosporus* was from the Indonesian Culture Collection, Research Center for Biology, the Indonesian Institute of Science, Cibinong Indonesia. Black CI rice (Bogor, West Java, Indonesia) and IPR30 rice was from the Indonesian Center for Rice Research, Indonesian Agency for Agricultural Research and Development, Ministry of Agriculture, Subang, West Java, Indonesia.

### 4.2. Sample Preparation

Two types of RB were used in this study. Black CI continued with the milling process using a Rice Machine-THU (Satake, Japan) to obtain brown rice. White IPR30 was in brown rice from. Two types of brown rice were processed by mini rice mill processing (Satake Grain Testing Mill, Hiroshima, Japan); then the RB was sieved as described previously [15]. *R. oligosporus* were maintained on potato dextrose agar media. The culture and fermentation process had been prepared as described previously [15]. For TPC and antioxidant analysis, fermented and non-fermented RB were extracted as described previously [13] with some modifications [15]. Fermented and non-fermented RB were extracted with methanol (HPLC grade) at 1:10 (*v*/*v*) by shaking in an orbital shaker at 30 °C (150 rpm) for 3 h and then sonicated for 10 min. The methanol-extracted samples were centrifuged at 7826× *g* for 10 min, and the supernatant was filtered. The filtrates (methanol extract) were stored at −20 °C until analysis. After the harvest, the RB was mixed with distilled water and centrifuged at 7826× *g* for 15 min; then the suspension was filtered and lyophilized.

### 4.3. Analysis of Total Phenolic Content (TPC) and Antioxidant Activity

TPC and antioxidant activity (2,2-diphenyl-1-picrylhydrazyl, DPPH assay) were determined by microplate methods described by [46] with slight modification. Twenty microliters of each sample were transferred into a 96-well plate then reacted with 100 µL of diluted Folin–Ciocalteu’s for TPC analysis and reacted with 180-µL working solution DPPH for antioxidant activity analysis. DPPH RSA values are expressed as mg TE per 100 g of sample DB.

### 4.4. GC-MS Analysis

Volatile compounds from two types of fermented and non-fermented RB (CI and IPR30) were extracted using the headspace-solid phase microextraction (HS-SPME) attached with divinylbenzene/carboxen/polydimethylsiloxane (DVB/CAR/PDMS) StableFlex fiber of 50-/30-µm thickness and 2-cm length (Supelco, Inc., Bellefonte, PA, USA) following Zeng et al. [20] with a slight modification. Three grams of sample and 0.4 µL 0.01% internal standard (IS) 2,4,6-trimethylpyridine (Sigma-Aldrich, Saint Louis, MO, USA) were transferred into a 22-mL headspace vial and covered with a silicone septum. Then the extraction fiber was inserted into the vial to be extracted in a water bath at a temperature of 80 °C for 30 min. After extraction, the fiber was removed from the vial and fed into the GC-MS injector at 250 °C hot desorption for 5 min. Every peak area in the chromatograms was standardized by the resulting area for the TMP peak. The GC-MS analysis was determined with GC-MS Agilent 7890A-5975C (Agilent Technologies, Palo Alto, CA, USA). Chromatographic separation was performed with an DB-WAX capillary column (30 m × 0.25 mm i.d. and 0.25-μm film thickness, Agilent, J & W) under the following instrumental conditions: helium as carrier gas at a constant flow of 0.8 mL/min, pressure of 60 kPa, electron ionization voltage of 70 eV, injector with mode splits at temperature 250 °C, and the oven initial temperature of 40 °C for 2 min, which was increased to 230 °C with 3 °C/min rate. Identification of the volatile components was based on a comparison of their mass spectra with those present in the NIST 2.0 database and confirmed by comparing their retention indexes with the published references [56]. Linear retention indexes (LRI) were calculated using the retention data of linear alkanes (C8–C30, Fluka) solution in n-hexane [20]. Relative amounts of volatiles were calculated by comparing their peak areas with IS peak area, whereby 5 μL IS are equal with 50 g sample. Data were analyzed as a mean of three replications.

### 4.5. Sensory Profile Analysis

Sensory profile was analyzed using projective mapping with 75 naïve panelists (based on their interest and availability) to evaluate the color, taste, flavor, and texture of the samples [52]. This study was based on ISO 13299:2016 Sensory Analysis—Methodology—General Guidance to establish a sensory profile. All panelists supplied informed consent before the examination. The preference mapping was used to evaluate which sample was preferable by the panelist as well as indicate the attribute related to preference [57]. Five samples with trivial code were used in this study: (1) 534 Benchmark (control)-a white rice bran derived from Ciherang cultivar, (2) 192 CI fermented RB, (3) 736 CI non-fermented RB, (4) 298 IPR30 fermented RB, and (5) 375 IPR30 non-fermented RB, respectively. Samples were prepared by the following procedure: 0.5 g of samples were mixed with 2.0 g commercial cereal and added to 15 mL of plain milk, then served to panelists. The panelists were free to place the sample in the 60 × 60 cm paper, based on their preference and similarity/dissimilarity of the sample with the benchmark.

### 4.6. Statistical Analysis

All data are reported as the mean and standard deviation. One-way analysis of variance using SPSS version 22.0 (SPSS, Inc., Chicago, IL, USA) was performed by two-way analysis followed by Duncan’s multiple range test for TPC and antioxidant parameters. The level of *p* < 0.05 was considered to indicate a significant difference between the means of groups. The preference mapping was analyzed by using multiple factor analysis (MFA) with Software R v.3.6.0. The MFA generates two figures simultaneously, which are Hierarchical Analysis and preference mapping. The PCA analysis was done by XLStat 2019 (New York, NY, USA).

## 5. Conclusions

In conclusion, this study analyzed and compared the TPC, antioxidant activities, volatile compounds, and sensory profiles of two RB cultivars before and after fermentation. It was observed that fermentation using *R. oligosporus* enhanced TPC and antioxidant activity of RB. Regarding antioxidant activity, future studies are needed to use another method. PCA plot analysis was located in different dimension; this means that the fermentation process can affect and differentiate the volatile compounds of RB, sensory profile, and the acceptance of the samples. The fermentation may amplify active compounds of RB and has the potential to produce functional ingredients for human health promotion.

## Figures and Tables

**Table 1:** Total phenolic content (TPC) and DPPH radical scavenging activity (RSA) of RB.

Incubation Time (h)	IPR30		CI	
	TPC	DPPH RSA	TPC	DPPH RSA
0	1.57 ± 0.19 ^a^	0.94 ± 0.13 ^a^	6.12 ± 0.70 ^a^	9.22 ± 0.77 ^a^
48	1.96 ± 0.02 ^ab^	1.21 ± 0.66 ^b^	6.10 ± 0.53 ^a^	5.27 ± 0.03 ^b^
72	2.24 ± 0.21 ^b^	1.46 ± 0.14 ^bc^	7.85 ± 0.61 ^b^	10.37 ± 0.82 ^ac^
96	2.18 ± 0.28 ^b^	1.80 ± 0.05 ^c^	7.34 ± 0.72 ^b^	11.74 ± 0.84 ^c^

TPC are expressed as [mg of gallic acid equivalents (GAE)/g DB and DPPH radical scavenging activity (RSA) are expressed as mg Trolox equivalent (TE) per 100 g of sample DB. Values are given as the means ± SD, n = 4. Means with the different letters within column are significantly different (*p* < 0.05) followed by Duncan’s multiple range test.

**Table 2:** Volatile compounds identified in fermented and non-fermented RB of IPR30 and CI.

Compounds	Code	LRI Experiment	LRI Reference	Relative Peak Area (µg/kg)				Aroma Description
				IPR30		CI		
				Non-Fermented	Fermented	Non-Fermented	Fermented	
**Alcohols**								
3-methyl-3-buten-1-ol	Al1	1252	1255 [36]	0.007 ± 0.0038 ^a^	0.03 ± 0.013 ^bc^	0.012 ± 0.005 ^ab^	0.07 ± 0.018 ^c^	sweet, fruity [38]
4-methyl-3-penten-1-ol	Al2	1392	1478 [39]	nd	nd	nd	0.044 ± 0.005	
2-butoxyethanol	Al3	1409	1379 [40]	0.003 ± 0.0029	nd	nd	nd	
2-ethylhexan-1-ol	Al4	1495	1491 [39]	0.001 ± 0.0003	0.004 ± 0.004	0.004 ± 0.003	0.004 ± 0.002	citrus, oily, citrus, greeny [41]
Linalool	Al5	1551	1553 [42]	nd	nd	0.003 ± 0.0007	nd	waxy, citrus, greeny [43]
1-Octanol	Al6	1564	1565 [42]	0.007 ± 0.0018	nd	0.003 ± 0.001	nd	fatty [43]
2,3-Butadienol	Al7	1584	1581 [44]	nd	0.048 ± 0.051	0.12 ± 0.018	0.09 ± 0.07	butter, creamy [41]
2-Furanmethanol	Al8	1663	1666 [36]	0.015 ± 0.0028 ^a^	0.009 ± 0.003 ^a^	0.018 ± 0.005 ^ab^	0.025 ± 0.005 ^b^	burnt, sweaty, floral [43]
Benzyl alcohol	Al9	1881	1879 [45]	0.006 ± 0.0021	0.056 ± 0.027	0.008 ± 0.004	0.016 ± 0.003	fruity, floral slightly [43]
Phenylethyl alcohol	Al10	1918	1871 [35]	0.005 ± 0.0069	0.01 ± 0.003	0.007 ± 0.009	0.011 ± 0.0005	rose [35]
Eugenol	Al11	2174	2171 [46]	0.002 ± 0.001	0.002 ± 0.002	0.002 ± 0.002	0.004 ± 0.005	sweaty, clove-like, greeny [46]
Nicotyl Alcohol	Al12	2236		nd	0.007 ± 0.002	nd	nd	
Glyserin	Al13	2329	2322 [47]	nd	0.065 ± 0.018	nd	0.024 ± 0.003	sweaty [43]
**Aldehydes**								
2-Propenal	Ad1	866	725 [39]	nd	0.011 ± 0.003	nd	0.006 ± 0.003	almond [43]
3-methylbutan-2-ol	Ad2	927	927 [44]	0.002 ± 0.0008	nd	0.008 ± 0.004	nd	malty, dark chocolate [36]
Hexanal	Ad3	1085	1093 [42]	0.011 ± 0.0066	nd	0.019 ± 0.00002	nd	grassy, tallow, fatty [36]
Octanal	Ad4	1297	1291 [45]	0.006 ± 0.0012	nd	nd	nd	fatty, greeny [48]
Nonanal	Ad5	1392	1397 [45]	0.019 ± 0.0048	nd	0.013 ± 0.005	nd	fatty, citrus, greeny [36]
Furfural	Ad6	1462	1471 [42]	0.005 ± 0.0006 ^bc^	0.002 ± 0.0008 ^a^	0.003 ± 0.0003 ^ab^	0.006 ± 0.002 ^c^	sweaty, caramel [43]
Benzaldehyde	Ad7	1520	1521 [49]	nd	nd	0.004 ± 0.002	0.007 ± 0.002	almond [43]
(E)-2-Nonenal	Ad8	1535	1542 [46]	0.001 ± 0.00002	0.0004 ± 0.0002	0.0004 ± 0.00007	0.0004 ± 0.0003	greeny, fatty [43]
Benzene acetaldehyde	Ad9	1639	1643 [49]	0.003 ± 0.001	0.004 ± 0.0007	0.01 ± 0.014	nd	sweaty [43]
4-Chlorobenzaldehyde	Ad10	1770		nd	nd	nd	0.005 ± 0.003	
Vanillin	Ad11	2577	2589 [50]	0.007 ± 0.0012	nd	0.004 ± 0.001	nd	vanilla [36]
**Ketones**								
Butenone	Kt1	952	932 [41]	nd	0.017 ± 0.006	nd	0.015 ± 0.011	caramel [40]
4-hydroxybutan-2-one	Kt2	1538		nd	0.002 ± 0.0007	nd	nd	
1-(1H-pyrrol-2-yl)ethanone	Kt3	1978	2017 [39]	0.001 ± 0.0002	0.002 ± 0.0006	0.001 ± 0.0003	0.002 ± 0.0006	nutty, walnut, bready [36]
2-Pyrrolidinone	Kt4	2056	2020 [51]	0.001 ± 0.0001	0.002 ± 0.0002	nd	0.0004 *	
6,10,14-Trimethyl-2-Pentadecanone	Kt5	2129	2110 [46]	0.004 ± 0.0012	0.003 ± 0.002	0.001 ± 0.0005	0.002 ± 0.0005	oily, herby [43]
**Acids**								
Acetic acid	As1	1450	1457 [42]	0.058 ± 0.0206 ^b^	0.012 ± 0.006 ^a^	0.022 ± 0.008 ^a^	0.108 ± 0.007 ^c^	sour [36]
Hexanoic acid	As2	1853	1853 [42]	0.0077 ± 0.0014	0.003 ± 0.0008	0.008 ± 0.002	0.004 ± 0.001	sweaty [36]
Heptanoic acid	As3	1960	1976 [39]	0.001 ± 0.0007	nd	nd	nd	
2-ethylhexanoic acid	As4	1958	1969 [39]	0.001 ± 0.0003	0.001 ± 0.00008	0.002 ± 0.0007	0.003 ± 0.001	
Octanoic acid	As5	2068	2067 [42]	0.005 ± 0.0005	0.003 ± 0.0006	0.004 ± 0.001	0.007 ± 0.001	sweaty, cheesy [36]
Nonanoic acid	As6	2175	2185 [45]	0.006 ± 0.0003	0.003 ± 0.0006	0.005 ± 0.002	0.008 ± 0.001	greeny, fatty [36]
**Esters**								
Methyl tetradecanoate	Es1	2012	2014 [42]	0.02 ± 0.0151	0.008 ± 0.0002	0.0004 ± 0.0001	0.005 ± 0.0007	fatty, waxy, oily [43]
Methyl hexadecanoate	Es2	2220	2203 [52]	0.003 ± 0.001	0.047 ± 0.002	0.001 ± 0.0004	0.027 ± 0.005	fatty, waxy, oily [43]
Methyl (E)-octadec-9-enoate	Es3	2451		0.003 ± 0.0012	0.027 ± 0.002	nd	0.009 ± 0.002	fatty, waxy, oily [43]
Methyl (9Z,12Z)-octadeca-9,12-dienoate	Es4	2500	2477 [52]	0.003 ± 0.0005	0.022 ± 0.002	nd	0.008 ± 0.002	fatty, waxy, oily [43]
**Benzene**								
Ethyl benzene	Bz2	1126	1122 [40]	0.003 ± 0.0005	nd	0.014 ± 0.006	0.031 ± 0.015	paint [48]
Naphtalene	Bz3	1739	1718 [49]	0.003 ± 0.002	0.006 ± 0.0007	0.012 ± 0.008	0.007 ± 0.001	
**Terpene**								
1R-.alpha.-Pinene	Tr1	1032	1035 [49]	nd	0.033 *	0.019 *	nd	alcoholic, herby [43]
Caryophyllene	Tr2	1595	1576 [42]	nd	0.028 ± 0.013	0.017 ± 0.007	0.035 ± 0.024	spicy [43]
Propylene Glycol	Tr3	1597	1599 [43]	nd	nd	nd	0.013 ± 0.006	alcoholic, herby [43]
**Phenol**								
2-Methoxyphenol	Fn1	1863	1877 [39]	0.006 ± 0.0031 ^a^	0.014 ± 0.004 ^a^	0.042 ± 0.014 ^b^	0.087 ± 0.013 ^c^	smoky, black rice like [48]
Phenol	Fn2	2009	1996 [52]	0.024 ± 0.192	0.008 ± 0.001	0.003 ± 0.001	0.012 ± 0.002	phenol l [48]
4-methylphenol	Fn3	2087	2031 [44]	nd	0.001 ± 0.0004	nd	nd	phenol, smoky [48]
4-ethenyl-2-methoxyphenol	Fn4	2202	2223 [41]	0.042 ± 0.0071 ^c^	0.002 ± 0.008 ^b^	0.01 ± 0.004 ^a^	0.01 ± 0.003 ^a^	Nutty [53]
**Furan**								
2-Pentylfuran	Fr1	1231	1231 [44]	0.004 ± 0.001	nd	nd	nd	greeny, beany, buttery [48]
2,3-Dihydro Benzofuran	Fr2	2398	2391 [49]	0.008 ± 0.0018	0.006 ± 0.002	0.002 ± 0.0006	nd	
**Lactone**								
Butyrolactone	Lk1	1626	1613 [44]	0.003 ± 0.0008	0.0004 ± 0.0004	0.003 ± 0.001	nd	creamy, fatty [43]
Pantolactone	Lk2	2038	2051 [54]	0.003 ± 0.0018	nd	nd	nd	cotton candy [36]
**Thiazole**								
Benzothiazole	Tz	1964	1948 [49]	0.002 ± 0.0005	0.003 ± 0.002	nd	0.001 *	gasoline, rubbery [48]
**Pyridine**								
3-methylpyridine	Prd	1300		nd	0.024 ± 0.018	nd	nd	greeny [43]
**Pyrazine**								
2-methylpyrazine	Prz	1267	1273 [36]	0.002 ± 0.0005	nd	nd	nd	roasty, nutty [55]

Presentation of data on the relative amount of compounds from the calculation of the average relative area of 3 replications ± SD; nd = no detection; * = components obtained only at 1 replication. Numbers on the same line and different letters show significantly different (*p* < 0.05). Linear Retention Index (LRI) literature is obtained from journal references were analyzed by DB-WAX column. Aroma descriptions are obtained from journal articles and flavor website.

## Data Availability

Data are contained within the manuscript.
